# Hemorrhage Occluder™ Pin to control life-threatening bleeding during the removal of an infected sacrocolpopexy mesh: A case report

**DOI:** 10.1016/j.crwh.2023.e00533

**Published:** 2023-08-08

**Authors:** Sara B. Papp, Shivani Gaitonde, Shadman Baig, Philippe E. Zimmern

**Affiliations:** a University of Texas Southwestern Medical School, 5323 Harry Hines Blvd, Dallas, TX 75390, USA; b Department of Urology, University of Texas Southwestern Medical Center, 5323 Harry Hines Blvd, Dallas, TX 75390, USA; c Department of Surgery-Vascular surgery, University of Texas Southwestern Medical Center, 5323 Harry Hines Blvd, Dallas, TX 75390, USA

**Keywords:** Sacrocolpopexy, Infected mesh, Complication, Hemorrhage, Hemorrhage Occluder™ Pin

## Abstract

Vaginal mesh exposures and infections are recognized complications of open and laparoscopic sacrocolpopexy performed for vault prolapse. In severe cases, complete sacrocolpopexy mesh removal may be necessary. This case report presents a 72-year-old woman with previous mesh sacrocolpopexy who presented with infected mesh and recurrent vaginal bleeding despite multiple attempts at surgical transvaginal mesh excision. A life-threatening massive hemorrhage occurred intra-operatively. After several failed attempts to control bleeding, hemorrhage Occluder™ Pins were successfully placed by vascular surgery to control presacral veins. Although an exceedingly rare complication, anticipation and rapid management of life-threatening bleeding are critical to save life during complicated mesh removals.

## Highlights

• Mesh infections are rare but serious complications of abdominal sacrocolpopexy.
• Complete removal of infected mesh may be necessary and carries high risks of complications.
• Sudden bleeding during infected mesh removal may require a multi-disciplinary approach for treatment.
• Hemorrhage Occluder™ Pin placement may be necessary to control presacral bleeding.

## 1 Introduction

Abdominal sacrocolpopexy (SCP) is a time-tested approach for pelvic organ prolapse (POP) repair with reported success rates of 78–100% [1]. The most frequent complications include stress incontinence, urinary tract infections, and mesh infection or exposure [2]. *Re*-operation rates for recurrent POP range from 0% to 18.2% between studies [1]. Vaginal mesh exposures are reported to occur in 3.4–9% of cases, most frequently presenting with vaginal bleeding and discharge [1,3,4]. For these cases, partial and complete transvaginal mesh removals are often necessary [5]. A new technique of holmium laser vaginoscopy was recently reported for mesh exposed at the apex of long and verticalized vaginal canals [6]. Depending on the location of the exposure and the extent of mesh infection, such removals may be difficult and carry high risks of complications [7].

This case report presents a completely infected SCP mesh requiring open removal. A sudden and cataclysmic bleed occurred from large presacral veins during surgery, prompting an emergency massive transfusion protocol and a vascular surgery consultation. Hemorrhage Occluder™ Pins were placed to control this bleeding and save the patient's life.

## 2 Case Presentation

A 72-year-old woman, gravida 4 para 3, presented to the emergency department with increased vaginal bleeding and worsening abdominal and back pain. The patient had a history of gastroesophageal reflux (GERD), chronic back pain, and recurrent urinary tract infections (UTI) as well as a past surgical history relevant for a cholecystectomy, total abdominal hysterectomy (TAH), bilateral salpingo-oophorectomy (BSO) for endometriosis in her late thirties, and a mesh sacrocolpopexy (SCP) with midurethral sling placement (MUS) at the age of 56. She underwent subsequent transvaginal mesh excision surgeries for recurrent vaginal bleeding at ages 57 and 59.

**Fig. 1:**
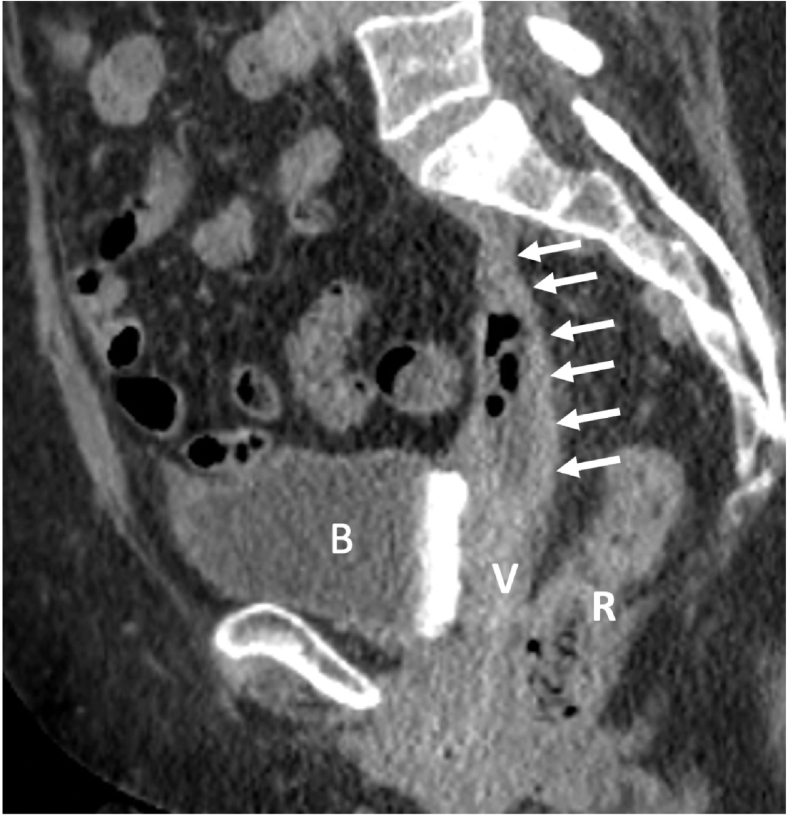
CT abdomen/pelvis sagittal view. CT abdomen/pelvis sagittal view showing infected mesh with fluid and gas collection attached over the sacrum. (B) bladder, (V) vagina, (R) rectum.

**Fig. 2:**
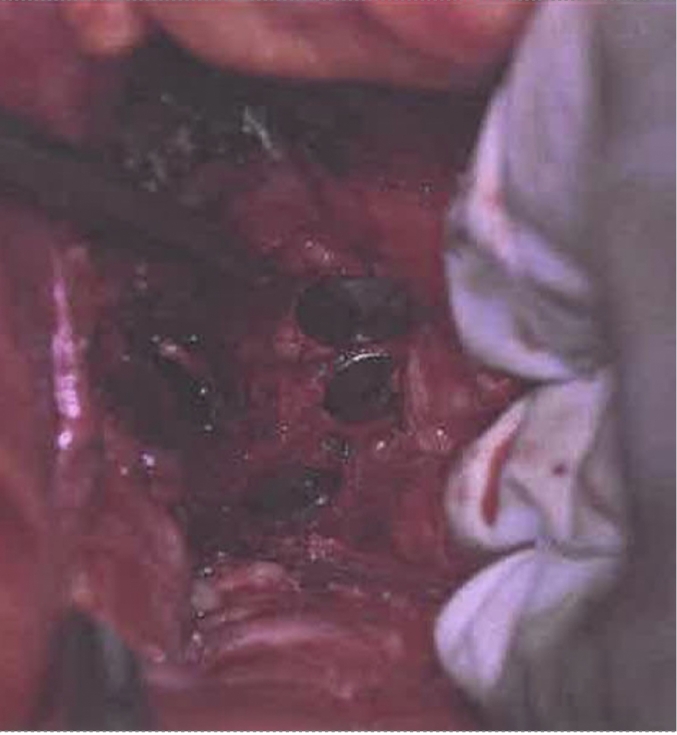
Intraoperative Hemorrhage Occluder™ Pin placement. Intra-operative pins placed by vascular surgery. Colon retracted by hand of operative surgeon seen on right.

**Fig. 3:**
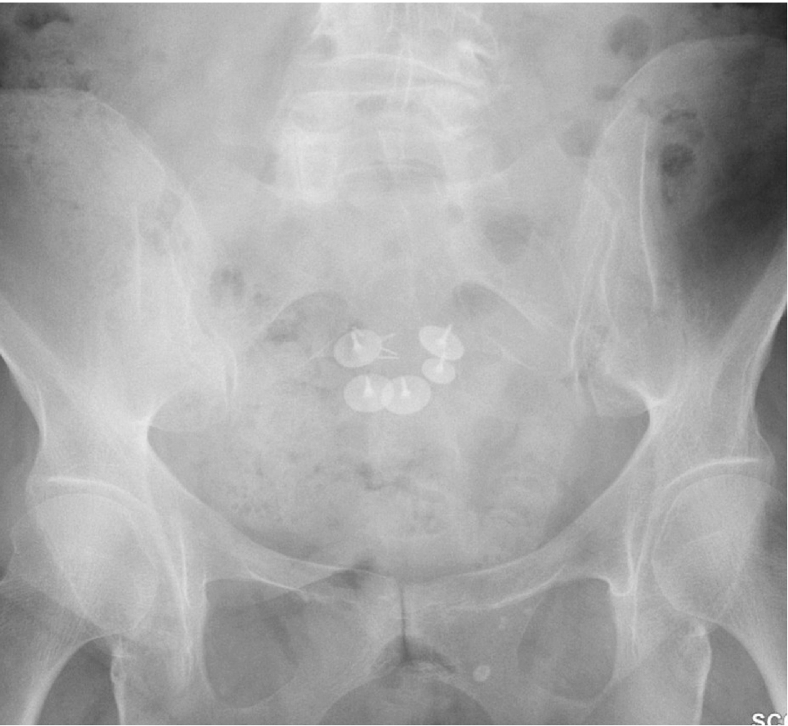
Post-operative X-ray of Hemorrhage Occluder™ Pin placement. Post-operative kidney, ureter, bladder (KUB) X-ray showing location of Hemorrhage Occluder™ Pins.

**Fig. 4:**
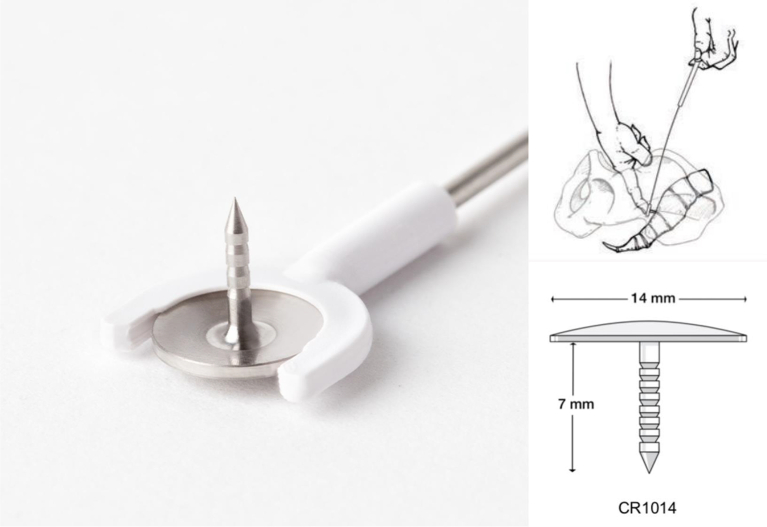
Hemorrhage Occluder™ Pin (Surgin). © 2023 Surgin | Hemorrhage Occluder™ Pin and Salgado Driver are trademarks of Surgin. | All rights reserved. Reproduced with permission.

**Fig. 5:**
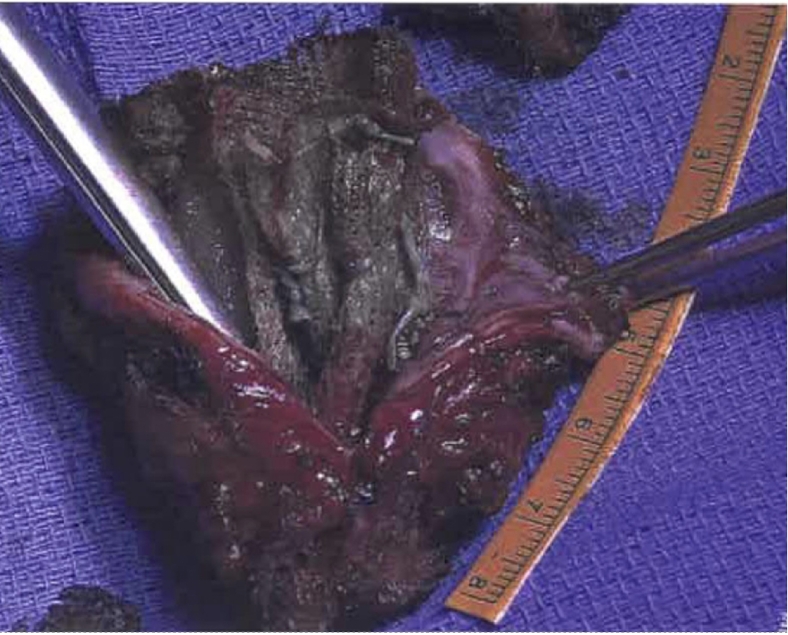
Specimen showing an abscess pocket surrounding the mesh. Removed specimen showing an abscess pocket surrounding the mesh with a thick capsule and copious amounts of purulent material.

Vaginal examination confirmed exposed mesh at the vaginal apex surrounded by easily bleeding granulation tissue. CT imaging revealed a fluid- and gas-containing collection alongside the course of the mesh all the way to the mesh attachment over the sacrum (Fig. 1). During laparotomy, the dissection detached the mesh-anchoring sutures from the promontory. Despite maintaining a gentle elevation of the infected mesh away from the sacrum, the surgical team encountered sudden brisk bleeding from large presacral veins. Anesthesia was immediately notified, and a vascular surgery consultation was initiated. Initial electrocautery attempts and suture ligation by the urgently contacted vascular team were unsuccessful. Packing and compression were performed to control this massive hemorrhage while several IV accesses were obtained, and a massive transfusion protocol was started. The decision was made to use 14 mm Hemorrhage Occluder™ Pins (Surgin) with TachoSil patches (fibrin sealant) to control this profuse venous bleeding (Fig. 2, Fig. 3, Fig. 4**).** The original procedure was resumed once hemostasis was achieved and the patient was stabilized. The infected mesh was completely removed down to the vaginal cuff exposure. The removed specimen showed an abscess pocket with a thick capsule and copious amounts of purulent material (Fig. 5). Intraoperative cystoscopy showed no bladder erosion. The surgical procedure lasted just over 4 h and required five units of blood transfusion and three units of fresh frozen plasma intra-operatively. The patient was initially monitored in the intensive care unit but discharged home three days later. Broad-spectrum intravenous antibiotics were administered while waiting for intra-operative cultures of the infected mesh to return speciation. The patient had an uneventful recovery with no prolapse or mesh exposure recurrence noted at her 6-month follow-up visit.

## 3 Discussion

This case report presents a rare and cataclysmic bleed during the open removal of a completely infected mesh placed several years previously for POP. The vaginally exposed mesh was responsible for recurrent vaginal bleeding episodes despite two excision attempts in the past. An open procedure was decided due to a very concerning fluid and gas collection seen on the CT scan as well as the patient's severe symptoms. This challenging intervention started with the detachment of the infected mesh from prior Ethibond sutures secured in the upper part of the sacrum. This unusual location (the anterior vertebral ligament above the disc space is traditionally recommended [8]) may have contributed to the development of large and compressed venous plexuses adjacent to the infected mesh graft material. Upon freeing this infected mesh with its surrounding thickened capsule, the friable vessels started to bleed profusely and broadly. The intensity of the bleeding was immediately recognized, and packing was performed. Anesthesia was alerted to obtain large-bore intravenous accesses to allow rapid blood transfusion while vascular surgery was consulted.

During pelvic trauma or colo-rectal surgery (proctectomy), bleeding can arise from important veins coursing on the surface of the sacrum or from the foramina of sacral basivertebral veins. A multidisciplinary approach requiring seven surgeons was necessary to stop presacral bleeding in the case of one colo-rectal surgery [9]. This type of bleeding can be dangerous and, at times, fatal [10]. The Hemorrhage Occluder ™ Pin was designed as a salvage technique to efficiently compress these large veins over the bony sacrum [11]. For the case outlined in this report, vascular surgery added a fibrin patch to broaden the surface of compression. Traditionally, the pin can be pushed in place by the Salgado driver. In the presented case, the presence of the infected mesh in the vicinity did not offer sufficient space; therefore, each pin was pressed down manually into the sacrum. A total of five pins were needed to control the bleeding (Fig. 2, Fig. 3, Fig. 4). The cataclysmic volume of blood loss from these large sacral and presacral venous plexuses can be overwhelming and rapid interventions such as packing and compression may not be sufficient to tamper this bleeding. All resources must be urgently activated in the operating room, including anesthesia, large venous accesses, blood transfusion protocol, vascular consultation, and identifying the location of these pins or tacks (as several may be needed at one time).

A review of the literature indicates rare prior reports especially in the past decade due to the rapid acceptance of robotic surgery for SCP during which venous bleeding can be easier to control. However, robotic technology is not yet available everywhere and certain re-operations are not amenable to a robotic approach.

In prior literature, Nygaard et al. reported a 4.4% rate of hemorrhage, blood transfusion, or both during abdominal sacrocolpopexy, while Rondini et al. found a 3.7% rate of overall intra-operative complications but did not differentiate between types of complications [1,12]. In a retrospective cohort study comparing various methods of sacrocolpopexy, Biler et al. found one case of abdominal sacrocolpopexy where intra-operative damage to the presacral veins resulted in massive hemorrhage [13]. In this case, a *Z*-suture along with a warm sponge compression were enough to stop the bleeding. The patient did not require a blood transfusion. The last abdominal sacrocolpopexy case report to date with intra-operative hemorrhage that required the use of pins was in 1991 by Timmons et al. [14] Several other case reports on various gynecological surgeries have required thumbtack placement when packing, ligating, clipping, or electrocauterization was unsuccessful [15,16].

## 4 Conclusion

The teaching point of this case report is to alert pelvic reconstructive surgeons to the availability of these Hemorrhage Occluder™ Pins designed to handle massive bleeding from sacral venous plexuses and to reinforce the importance of a multi-modality approach to achieve hemostasis and save life.
